# Drug-induced ectropion following the chronic use of topical Natamycin

**DOI:** 10.1186/s12348-020-00230-2

**Published:** 2020-12-21

**Authors:** Mohammad Soleimani, Farzad Pakdel, Mohammad Mehrpour

**Affiliations:** Ocular Trauma and Emergency Department, Farabi Eye Hospital, Tehran, Iran

**To the Editor.**

Drug-induced ectropion is a rare condition secondary to drug hypersensitivity. It was reported following administration of topical and systemic medications [1–7]. Among topical drugs, anti-glaucoma medications were the most reported drugs causing periorbital dermatitis and ectropion; however, other topical agents such as topical 5% fluorouracil cream and topical Tretinoin were described in the literature [4, 5]. Early cessation of offending medication was mentioned as the most important prognostic factor to reverse this complication without surgical intervention in previous studies [8].

Topical 5% Natamycin is the empirical topical medication for the treatment of fungal keratitis [9]. We reported drug-induced ectropion following chronic use of Natamycin in this study. To the best of our knowledge, there was no study in the literature reporting ectropion as a complication of Natamycin.

## Case presentation

**Fig. 1:**
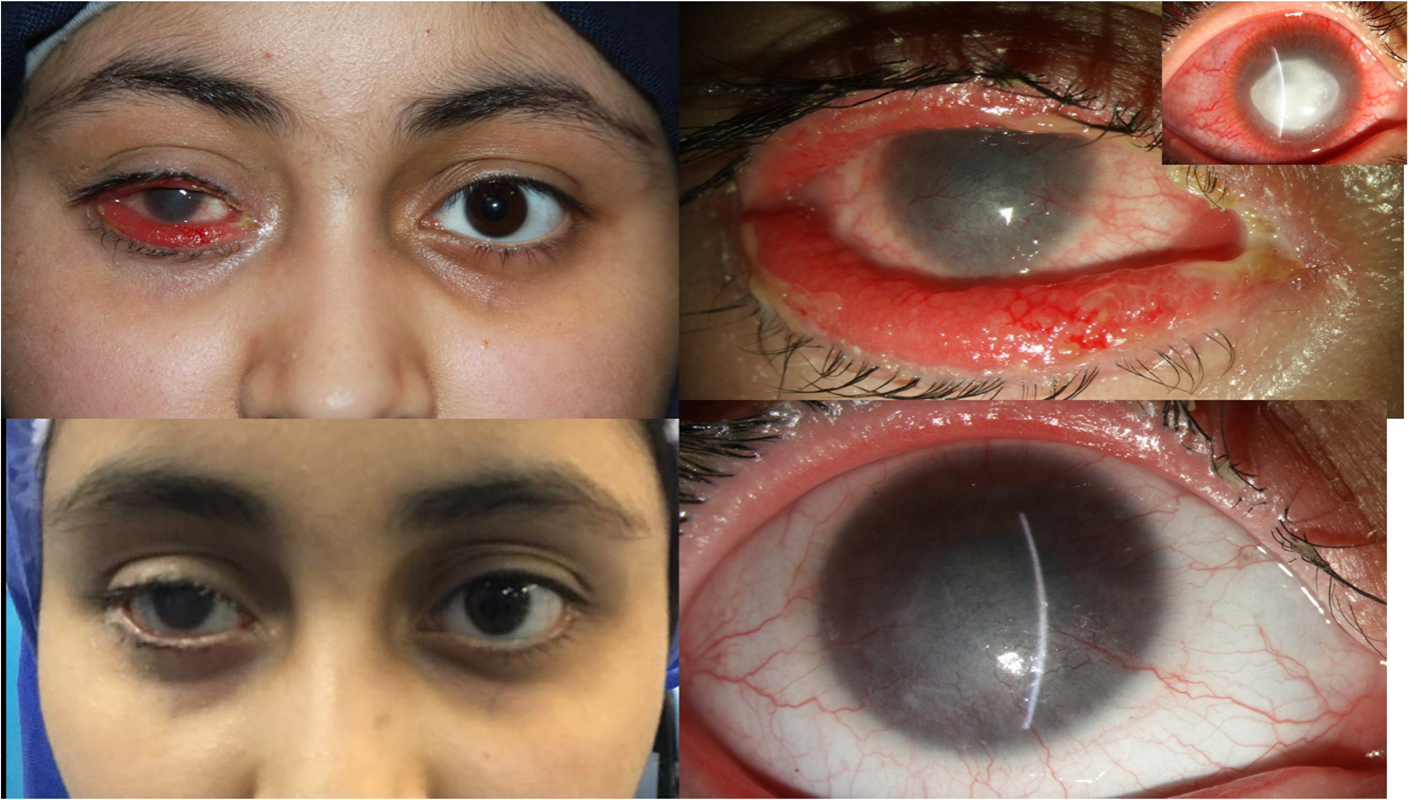
**a** and **b** show ectropion and papillary conjunctival reaction after chronic use of Natamycin for fungal keratitis (**c**). **d** and **e** show resolution of right eye ectropion after cessation of topical Natamycin

A 15-year-old lady presented with symptoms of allergic conjunctivitis such as epiphora, pain, and redness of the lid margin of the right eye. On her eye examination, she had visual acuity of counting finger from 1 m, diffuse vascularized corneal opacity, ectropion and injected conjunctiva with papillary reaction. Additionally, she had physical signs of allergy such as periorbital edema, erythema and scaling of adjacent skin (Fig. 1a, b). However, there were not any signs of cicatricial component such as lamellar shortening or lid retraction. She had a history of admission in our ward for the same eye fungal keratitis 9 months ago (Fig. 1c). She had been treated with topical Natamycin (Natacyn, Alcon, Fort Worth, TX). After partial improvement of the corneal ulcer, she had been discharged with topical 5% Natamycin four times a day. She hadn’t come for follow up visits and continued using Natamycin for a total of 9 months until her recent visit. She did not use any other topical or systemic medication during that time. We stopped Natamycin and administered lubrication and a topical steroid (0.1% Fluorometholone (FML, Allergan, Inc., Irvine, CA) every 6 h. One month later, the ectropion resolved completely (Fig. 1d, e).

## Discussion

To the best of our knowledge, we reported the first case of ectropion induced by topical Natamycin.

Natamycin is a fungicidal tetraene polyene antibiotic; it has been reported as the most effective medication against Fusarium and Aspergillus [9]. Allergic reactions such as eyelid edema, conjunctival hyperemia and irritation were reported as common side effects of Natamycin [10].

The pathophysiology of drug-induced ectropion was described as a two-phase process. First, the allergic reaction results in tissue edema which can exacerbate lid laxity and cause mechanical ectropion; however, lid laxity was not detected in our patient. Second, chronic hypersensitivity leads to cicatrization causing anterior lamellar shortening and cicatricial ectropion [8]. Delay in stopping responsible medication and establishment of cicatrization decrease the rate of spontaneous resolution of ectropion [1, 8]. However, in our case, in spite of 9 months use of natamycin, the cessation of drug administration improved ectropion entirely.

Hegde et al. investigated 13 patients with drug-induced ectropion. Dorzolamide followed with brimonidine were the most offending medications in their study. They concluded that all patients who had been managed with stopping responsible drug and a short course of steroid therapy did not need corrective surgery which was consistent with our experience [8]. In other studies, topical dipivefrin, apraclonidine, fluorouracil, and Tretinoin were reported as causative agents of ectropion [2–5]. In addition, the systemic use of epidermal growth factor receptor (EGFR) inhibitors such as cetuximab and erlotinib was reported as a cause of reversible cicatricial ectropion [11]. In contradiction to these studies whose cases were elderly patients considered to have some degree of involutional ectropion, our case was young. It should be noted that the above-mentioned side effect could be related to benzalkonium chloride 0.02% (the preservative in Natacyn); however, the concentration is not significant and despite wide-spread use in different ophthalmic drops, there is not any report regarding this complication.

## Conclusion

This is an interesting case report illustrating natamycin causing reversible ectropion. Usually the most difficult part is isolating the offending allergy, but given the patient’s history of only using natamycin, authors are able to pinpoint natamycin as the culprit.

## Data Availability

Our data are available in medical records of Farabi Eye Hospital.

## Abbreviation

EGFR: Epidermal growth factor receptor
